# Is the association between self-rated health and underlying biomarker levels modified by age, gender, and household income? Evidence from Understanding Society – the UK Household Longitudinal Study

**DOI:** 10.1016/j.ssmph.2019.100406

**Published:** 2019-05-08

**Authors:** M.Pia Chaparro, Amanda Hughes, Meena Kumari, Michaela Benzeval

**Affiliations:** a Institute for Social and Economic Research (ISER), University of Essex, Colchester, Essex, United Kingdom; b MRC Integrative Epidemiology Unit, Bristol Medical School, University of Bristol, United Kingdom

**Keywords:** Self-rated health, Biomarkers, Age, Gender, Income, United Kingdom, Health inequalities

## Abstract

The goal of this study was to evaluate how self-rated health (SRH) and objective measures of health (biomarkers) are associated, and if this association varies by gender, age, and socioeconomic position (measured by household income). Data come from the UK Household Longitudinal Study nurse visit (2010–2012), including a representative sample of adults in Great Britain (N = 15 687 maximum sample). SRH was assessed by the question *“In general, would you say your health is excellent, very good, good, fair, or poor?”* and dichotomized into good or poor. Indices were created for four biomarker categories based on the aspects of health they are likely to reflect, including *visible weigh-related*, *fitness*, *fatigue*, and *disease risk* biomarkers. Logistic regression models were run with SRH as the outcome and each biomarker index as a predictor, adjusting by gender, age, and income. Further, interaction terms between each biomarker index and gender, age, and income (independently) were added to test for effect modification. All biomarker indices were associated with SRH in expected directions, with the *fitness* index most strongly predicting SRH. Gender, age, or income modified the associations between SRH and all biomarker indices to different extents. The association between the *visible weight-related* biomarker index (including body mass/fat variables) and SRH was stronger for women than men and for those in higher income groups than lower income groups. Income also modified the association between SRH and the *fitness* biomarker index, whereas age modified the association between SRH and the *fatigue* biomarker index. When using SRH to investigate health inequalities, researchers and policy makers should be clear that different social groups may systematically consider different dimensions of health when reporting their SRH.

## Highlights

• The association between self-rated global health and underlying objective health as measured by biomarkers varies by type of biomarker, age, sex and socioeconomic status.
• Biomarkers that measure different aspects of *fitness* most strongly predicting self-rated health.
• The association between the *visible* biomarkers (including body mass/fat variables) and self-rated health was stronger for women than men and for those in higher income groups than lower income groups.
• Income also modified the association between self-rated health and biomarkers of *disease*, with a stronger association for higher income groups.
• When rating own health individuals from different social groups may systematically consider different dimensions of health.

## Introduction

Self-rated health (SRH) is the most widely used proxy for health status in medical sociology research (Jylhä, 2009). Usually assessed with one question (e.g. “Overall, how would you rate your health?”), SRH is easy and cheap to collect and has been consistently linked to objective health measures (Christian et al., 2011; Jylhä, Volpato, & Guralnik, 2006; Leshem-Rubinow et al., 2015; Shanahan, Bauldry, Freeman, & Bondy, 2014; Tanno et al., 2012) and mortality (Idler & Benyamini, 1997; Lima-Costa, Cesar, Chor, & Proietti, 2012a). However, research has shown that the way people interpret and respond to the SRH question varies by age (Shooshtari, Menec, & Tate, 2007), gender (Benyamini, Leventhal, & Leventhal, 2000; Jylhä, Guralnik, Ferrucci, Jokela, & Heikkinen, 1998), and socioeconomic position (SEP) (Dowd & Zajacova, 2010). Jylhä et al. (Jylhä et al., 1998) highlights that several dimensions may be considered when rating one's health – including medical and nonmedical factors – but “(SRH) has to be understood as a summary measure of all the dimensions of health that are relevant *to the individual respondent* (emphasis added)” and, therefore, highly variable. Given the ubiquity of SRH in medical sociology research, public health policy and practice, it is crucial to understand how the meaning of SRH may vary by population sub-groups and the consequences of this for studying health inequalities.

Using data from *Understanding Society* – the UK Household Longitudinal Study (UKHLS), the objectives of this study were to: 1) evaluate how responses to SRH vary by underlying biomarker levels, and 2) evaluate if the association between SRH and biomarkers is modified by age, gender, and SEP (measured by household income). Biomarkers are objectively-measured markers of physiological systems which include physical measures (e.g. weight, height, blood pressure) and blood analytes (e.g. hemoglobin, c-reactive protein). Whereas previous research linking SRH with biomarkers levels has treated biomarkers as subclinical values/conditions of which participants are mostly unaware (e.g. Jylhä et al., 2006), this “invisibility” is not necessarily true. Biomarkers can also reflect visible health conditions, for example obesity. In addition, even if the respondent does not know their exact value for a given biomarker, they may feel symptoms of underlying diseases (e.g. fatigue) or may have a doctor's diagnosis of a health condition (e.g. diabetes, high blood pressure) (Idler & Kasl, 1991; Jylhä, 2009). We have grouped biomarkers into four categories (*visible weight-related*, *fitness*, *fatigue*, and *disease risk*) reflecting different ways they may make the respondent *feel* and hence assess their health, and assessed whether their association with SRH is modified by age, gender, and/or SEP.

## Background

In 2009, Jylhä developed a conceptual framework to understand the thought processes and decisions involved when answering the SRH question (Jylhä, 2009). Jylhä argues that there are different stages involved in the process of self-assessment. When asked to rate their own health, a person begins by assessing *health* as a concept: what does it mean for them and which health components are most relevant to them (Jylhä, 2009). This conceptualization is influenced by contextual and cultural factors, including the individual's demographic and social characteristics. In the second stage, individuals put their health in perspective considering the individual's life stage and also compared to peers: how is my health considering my age, previous health status and expected future, how does it compare to that of peers based on age, gender, socioeconomic condition, etc. (Jylhä, 2009). This stage can be influenced not only by who the individuals choose as their reference groups, but also by their mental state and their positive vs. negative disposition. Finally, individuals decide how to fit these decisions into the proposed self-rated scale; this in turn is influenced by cultural conventions in expressing positive vs. negative opinions (Jylhä, 2009). Therefore, it is expected that people's responses to the SRH question will vary not only by age, gender, and SEP, but also by cohort, culture, and social networks. Given these complexities, Jylhä (2009) argues that SRH should be understood as both a subjective and contextual self-assessment, and as an indicator of somatic and mental state.

Previous to Jylhä’s conceptual model, other researchers such as Blaxter (1990) and van Dalen, Williams, and Gudex (1994) made efforts to understand the decisions made when one rates their own health, including different definitions of health for subgroups of age, gender, and SEP. By using a series of qualitative and quantitative questions, their research points to different dimensions of health – including functionality, fitness, absence of illness, and psychosocial factors – having varying importance for specific population subgroups (Blaxter, 1990; van Dalen et al., 1994). Moreover, people's responses vary if defining health for oneself vs. others (Blaxter, 1990; van Dalen et al., 1994).

Blaxter's research was based on *The Health and Lifestyle Survey*, a national survey of adults aged >18 years in England, Wales and Scotland in 1984–1985 (Blaxter, 1990). Among other things, survey participants were asked two sets of open-ended questions regarding their definition of health: (1) “Think of someone you know who is very healthy. Who are you thinking of? How old are they? What makes you call them healthy?” and (2) “At times people are healthier than at other times. What is it like when you are healthy?” Blaxter found that responses differed by participant age, gender, and SEP. Among younger people, *physical fitness* seemed to be the most important definition of health, whereas it was the least favored concept for participants aged over 60, both when defining health for oneself or others. Younger men, in particular, also spoke of health in terms of *physical strength* and *fitness,* whereas women discussed physical fitness in terms of outward appearance, with *being or feeling slim* used to describe a healthy person. On the other hand, *energy* was the word most frequently used by all women and older men when describing health. Health as *function* was more often mentioned among older people. Health as *not being ill* was most commonly used to describe health in others, and more commonly mentioned for those with higher education and income. Among working-age men, particularly those who did manual work, health was often defined as *being able to do hard work*. Interestingly, at all ages but particularly among the elderly, those who were suffering from chronic conditions were less likely to define their health in terms of (lack of) illness. As Blaxter points out, “if illness symptoms are a taken for granted experience, or disease is seen as the norm, then health has to be defined in other ways” (Blaxter, 1990, pp. 21–22).

Regarding socioeconomic differences in SRH, Adams and White (2006) used data from the 1998 Health Survey for England to evaluate if the association between SRH and objective health measures (i.e. systolic blood pressure and body mass index (BMI)) varied by occupational social class. They found that, within the “very good or good” SRH category, those in the manual social class (compared to non-manual) were more likely to have higher blood pressure and BMI, with no such difference in the “less than good” SRH group; this suggested, the authors concluded, that the way people rank their health varies by social class (Adams & White, 2006). Similarly, Dowd and Zajacova (2010) found that the association between SRH and biomarkers varied by educational attainment among a nationally representative sample of US adults, with more pronounced educational differences in biomarker levels among those rating their health as “excellent.” Similar findings have been reported when looking at the predictive power of SRH for mortality, with a lower predictive power for those in disadvantaged socioeconomic groups and a graded difference in the strength of the association between the three socioeconomic groups studied (Lima-Costa et al., 2012b).

More recent studies also highlight that the way different subgroups rate their health may change over time. For example, the rise in both obesity prevalence and ubiquity of health messages focused on weight-related healthy behaviors (e.g. fruit and vegetable consumption, emphasis on physical activity) may affect the way people perceive and rank their health (Altman, Van Hook, & Hillemeier, 2016). Altman et al. (2016) argue that more recent cohorts would be more likely to take into account their own weight status *negatively* when ranking their health. However, this is not necessarily true as increases in obesity over time may lead to a higher social acceptability of larger body types (Burke, Heiland, & Nadler, 2010; Langellier, Glik, Ortega, & Prelip, 2014).

Based on previous research, in particular that of Blaxter (1990), we anticipated that the association between SRH and biomarkers will vary based on the type of biomarker under consideration, as well as by gender, age, and SEP. Therefore, we grouped available biomarkers into four categories based on the aspects of objective health they are likely to reflect, grounded on previous literature as well as on the authors' previous experience with biomarker data. The four categories included (see also Table 1): *visible weight-related* biomarkers (to self and others; called *visible* hereafter for simplification), including all biomarkers related to weight and body mass/fat (body mass index, waist circumference, and % body fat); *fitness* biomarkers, including heart rate, grip strength, and lung function variables; *fatigue* biomarkers, including c-reactive protein, fibrinogen, hemoglobin, ferritin, and cytomegalovirus infection; and *disease risk* biomarkers, including biomarkers that may be markers of underlying conditions (blood pressure, lung function, cholesterol and triglycerides, and glycated hemoglobin [HbA1c]).

**Table 1:** Use/purpose, measurement method, data management and operationalization of the biomarkers included in the study.^a^

Included biomarkers	Use/purpose	Measurement method	Data management & exclusions	Operationalization
**Visible (to self and others)**				
Body mass index (BMI, kg/m^2^)	To estimate weight status	Weight measured with Tanita BF 522 digital floor scale; height measured with a portable stadiometer	Exclusions: <18 years of age; implausible BMI (<15 or >60)	BMI, continuous Weight status: BMI<18.5 = underweight; BMI between 18.5 and 24.99 = normal weight; BMI between 25 and 29.99 overweight; BMI>30 = obese
Waist circumference (WC, cm)	To assess central adiposity	Tape with insertion buckle; measurement taken at the midpoint between the lower rib and the upper margin of the iliac rest. Average of two measurements	Exclusions: implausible WC (<50 or >190 cm; no implausible values found in the data)	Waist circumference, continuous Abdominal obesity: WC > 102 cm for men and >88 cm for women = abdominal obesity; WC between 94 and 102 cm for men and between 80 and 88 cm for women = at risk of abdominal obesity; WC < 94 for men and WC < 80 for women = normal
%Body fat (%BF)	To assess adiposity	Bioelectrical impedance using a Tanita BF 522 scale; estimation based on respondent's age, gender and height (a “standard” body type was assumed)	Exclusions: implausible %BF (<5%; no implausible values found in the data)	%Body fat, continuous Obesity: %BF>30% for women and >25% for men = obesity; %BF ≤ 30% for women and %BF ≤ 25% for men = no obesity
**Fitness**				
Heart rate (HR, bpm)	To assess resting heart rate	Portable monitor Omron HEM 907. Measured as the number of beats per minute; average of three readings	Exclusions: top 99.5 (≥102 bpm) and bottom 0.5 (≤44 bpm) percentiles	HR, continuous
Grip strength (GS, kg)	To measure muscular strength	Smedley dynamometer. Three measurements taken with each hand; only the maximum reading for dominant hand used	Exclusions: top 99.5 (≥66 Kg) and bottom 0.5 (≤7 Kg) percentiles	GS, continuous
Forced expiratory volume in 1 s, percent predicted (FEV1%)^b^ Forced vital capacity, percent predicted (FVC%)^b^	To assess lung function	NDD Easy On-PC spirometer (England and Wales only).	Data cleaning procedures: 1) if the reading for FVC was equal to the reading of FEV, these readings were considered invalid and set to missing; 2) if the ratio of FEV to FVC was >0.95, the FVC reading was considered invalid and set to missing Percent predicted FVC (FVC%) and FEV1 (FEV1%) were estimated from valid readings of FVC and FEV adjusting for age, gender, height and ethnicity following the European Respiratory Society Global Lung Function Initiative (ERS-GLI) equations (Quanjer et al., 2012). Exclusions: implausible values (<20% or >200%)	FEV1% and FVC%, continuous
**Fatigue**				
C-reactive protein (CRP; mg/L)	Chronic inflammation marker	Analyzed from serum using the N Latex CRP mono immunoassay on the Behring Nephelometer II Analyzer (Dade Behring, Milton Keynes, UK)	Exclusions: CRP>10 mg/L as these reflect a current infection instead of chronic inflammation (N = 705)	CRP, continuous
Fibrinogen (g/L)	Chronic inflammation marker	Analyzed from citrate plasma samples using a modification of the Clauss thrombin clotting method on the IL-ACS-TOPS analyzer	Exclusions: top 99.5 (≥4.8 g/L) and bottom 0.5 (≤1.4 g/L) percentiles	Fibrinogen, continuous
Hemoglobin (Hb, g/L)	Indicator of iron status	Measured from whole blood samples with a spectrophotometric assay on Sysmex XE-2100 analyzer	Exclusions: top 99.5 (≥17.1 g/L) and bottom 0.5 (≤9.6 g/L) percentiles	Hb, continuous Anemia: Hb < 130 g/L for men and Hb < 120 g/L for women = anemia; Hb ≥ 130 g/L for men and Hb ≥ 120 g/L for women = no anemia
Ferritin (μg/L)	Indicator of iron stores	Measured from serum samples with an electrochemiluminescent immunoassay on the Roche E170 analyzer	Exclusions: top 99.5 (≥822 μg/L) and bottom 0.5 (≤8 μg/L) percentiles	Ferritin, continuous
Cytomegalovirus antibody measurement (CMV)	To assess immunoscience (susceptibility to infection)	Two CMV antibodies measured: immunoglobulin G (IgG) indicating a past CMV infection, and immunoglobulin M (IgM) indicating a recent/current infection. Both measured from serum samples with an electrochemiluminescent immunoassay on the Roche E170 analyzer. For people with a positive/indeterminate IgM test, a confirmatory assay was conducted with an avidity test on the Mini VIDAS immunoassay analyzer		Categorized as ever having CMV infection vs. not
**Disease**				
*Blood pressure* Systolic blood pressure (SBP, mmHg) Diastolic blood pressure (DBP, mmHg)	Blood pressure, risk for heart disease	Portable monitor Omron HEM 907. Mean of three valid readings used	Exclusions: implausible SBP and DBP (<40 or >300 mmHg and <30 or >200 mmHg, respectively; no implausible values found in the data)	SBP and DBP, continuous
*Blood lipids* Total cholesterol (mmol/L) High-density lipoprotein (HDL) cholesterol (mmol/L) Triglycerides (mmol/L)	To assess fat in the blood, risk for heart disease	Total cholesterol, HDL and triglycerides were measured from (non-fasting) blood serum using enzymatic methods with a Roche Modular P analyzer. Cholesterol measures were calibrated to the Center for Disease Control guidelines	Exclusions: top 99.5 and bottom 0.5 percentiles Total cholesterol: ≤2.8 & ≥8.9 mmoL/L HDL: ≤0.7 & ≥3.1 mmoL/L Triglycerides: ≤0.5 & ≥7.3 mmoL/L	Total cholesterol, HDL, and triglycerides continuous
Glycated hemoglobin (HbA1c, mmol/mol)	Marker of undiagnosed or poorly managed type II diabetes	Measured from whole blood using HPLC cation exchange on a Tosoh G8 analyzer	Exclusions: top 99.5 (≥86 mmoL/mol) and bottom 0.5 (≤26 mmoL/mol) percentiles	HbA1c, continuous

a For more detailed information please see (Benzeval et al., 2014; McFall et al., 2014).

b Also included in the *disease* category.

We hypothesized that gender, age, and SEP will modify SRH- biomarker associations within the four biomarker groups as follows:

(1) The association between SRH and biomarkers for women, compared to men, will be: a) stronger for v*isible* biomarkers, b) weaker for *fitness* biomarkers, and c) stronger for *fatigue* biomarkers.
(2) The association between SRH and biomarkers for older people, compared to working-age adults, will be: a) weaker for *fitness* biomarkers, b) stronger for *fatigue* biomarkers, and c) weaker for *disease risk* biomarkers.
(3) The association between SRH and biomarkers for people with lower SEP, compared to higher, will be: a) weaker for *visible* biomarkers, b) stronger for *fitness* biomarkers, c) stronger for *fatigue* biomarkers, and d) weaker for *disease risk* biomarkers.

## Methods

### Data source

Data for this analysis comes from *Understanding Society*: the UK Household Longitudinal Study (UKHLS; https://www.understandingsociety.ac.uk/; University of Essex, 2015), which began in 2009 and includes a number of different samples (Knies, 2015). Specifically, this project is based on data from the General Population Sample (GPS) and British Household Panel Survey (BHPS) samples in waves 2 and 3 (2010–2012) respectively, which additionally included data from a Nurse Health Assessment (Knies, 2015; University of Essex, 2014). Detailed information on data collection procedures for UKHLS and the Nurse Health Assessment can be found elsewhere (Benzeval, Davillas, Kumari, Lynn, & Institute for Social and Economic Research (ISER), 2014; Knies, 2015; McFall, Petersen, Kamiska, Lynn, & Institute for Social and Economic Research (ISER), 2014).

The Nurse Health Assessment took place approximately 5 months after the main interviews for waves 2 or wave 3 from 2010 to 2012; eligibility criteria for participation included completion of the most recent main interview in English; being 16 or older; and not being pregnant (McFall et al., 2014). Only participants living in England, Wales, and Scotland were included as nurse recruitment proved difficult in Northern Ireland. In the second year of interviewing, only 81% of the GPS sample were randomly selected to take part due to shortages of qualified nurse interviewers. UKHLS participants fulfilling eligibility criteria were contacted via post and telephone calls to set up a home visit with the nurse. At the time of the visit, the nurse explained the protocol for health measures and blood sample collection and provided participants with an oral and written consent forms, respectively. Participants could decline any procedure or measurement at any time. During the nurse visit, participants received feedback on their anthropometric and blood pressure measures. Blood samples were not analyzed at the time and hence no feedback from them was possible All participants also received a £10 voucher upon completion of the nurse visit, as a thank you for participating.

From an eligible sample of 35 875 participants, 20 644 completed the health measures (57.5% response rate) and 13 517 provided blood (37.7% response rate) (Benzeval et al., 2014; McFall et al., 2014); subsequently blood samples were analyzed for a range of key analytes, with sample size further varying by each biomarker measure (Table 1).

### Data management

This analysis is cross-sectional, so all variables are taken from the same wave *for a given participant* – wave 2 (GPS participants) or wave 3 (BHPS participants). Our dependent variable was SRH, assessed by the question *“In general, would you say your health is excellent, very good, good, fair, or poor?”* SRH was dichotomized into *good* (including those who responded excellent, very good, or good) and *poor* (included those who responded fair or poor). Our independent variables included most biomarkers assessed in UKHLS, including a range of disease markers and body function measures, as listed in Table 1. Protocols and procedures related to these measurements can be found in detail elsewhere (Benzeval et al., 2014; McFall et al., 2014), with a summary of their use, measurement method, exclusion criteria, and operationalization included in Table 1.

Indices were created for each of the four biomarker groups by classifying individuals into sex-specific tertiles for each of the included variables (Table 2) and then summing the scores, whereby higher scores indicate worse health outcomes. For example, within the *visible* biomarker group, individuals were assigned as being in the lowest, middle, or highest (sex-specific) tertile for each of the variables included – BMI, waist circumference, % body fat – and the scores were then summed, with an index ranging from 3 (lowest tertile on all variables) to 9 (highest tertile on all variables). For biomarker groups where higher values of certain variables indicate better health outcomes (e.g. grip strength in the *fitness* group), reverse coding was applied.

**Table 2:** Cut-off points for sex-specific tertiles for the creation of the *visible weight-related*, *fitness, fatigue* and *disease risk* indices.

	Women		Men	
	Lowest tertile cut-off	Highest tertile cut-off	Lowest tertile cut-off	Highest tertile cut-off
**VISIBLE WEIGHT-RELATED BIOMARKERS**				
Body Mass Index (kg/m^2^)	24.9	29.8	26.0	29.7
Waist circumference (cm)	82.7	95.4	94.3	105.0
%Body fat (%BF)	33.7	40.9	19.6	27.3
**FITNESS BIOMARKERS**				
Heart rate (bpm)	65.0	73.5	62.0	71.5
Grip strength (kg)	24.0	30.0	38.0	47.0
FEV1%	87.5	100.4	85.7	98.9
FVC%	92.2	104.5	90.7	103.1
**FATIGUE BIOMARKERS**				
C-reactive protein (mg/L)	0.9	2.4	0.8	2.0
Fibrinogen (g/L)	2.6	3.0	2.5	2.9
Hemoglobin (g/L)	127.0	135.0	141.0	150.0
Ferritin (μg/L)	50.0	103.0	114.0	202.0
**DISEASE RISK BIOMARKERS**				
SBP (mmHg)	114.5	129.5	123.5	135.5
DBP (mmHg)	68.0	77.0	69.5	79.0
FEV1%	88.1	101.0	87.0	100.3
FVC%	92.9	104.9	92.1	104.4
Total cholesterol (mmol/L)	5.0	5.9	4.9	5.9
HDL cholesterol (mmol/L)	1.5	1.9	1.2	1.5
Triglycerides (mmol/L)	1.1	1.7	1.4	2.2
HbA1c (mmol/mol)	34.0	38.0	35.0	38.0

Gender (women vs. men), age, and household income, as a marker of SEP, were also included in the analysis to assess their potential moderation of the SRH – biomarker associations. Analyses were restricted for those age 25 years and higher so that most individuals will have completed education. For moderation analysis, age was categorized as working-age (25–60 years) vs. retirement-age (>60 years; 95th percentile = 80 years, highest value = 102 years). Household monthly net equivalised income (i.e. adjusted by household size using OECD scale, in £) was categorized in tertiles based on sample distribution. Educational attainment standardized by age and categorized in tertiles was also considered as a measure of SEP and assessed in moderation analysis. However, since the results with education were very similar to those obtained with income, we only present results with household income as a marker for SEP.

### Statistical analysis

All analyses were carried out in SAS v9.4 using sample weights to adjust for the complex sample design and likelihood of being included in the Nurse Health Assessment (Benzeval et al., 2014; Knies, 2015). Descriptive statistics were used to characterize the sample. The association between SRH (dependent variable) and each biomarker (independent variables), as well as the four biomarker indices, was assessed using logistic regression models while adjusting for gender, age (continuous), and household income.

To assess if the association between SRH and each biomarker group (i.e. *visible, fitness, fatigue,* and *disease risk*) was modified by gender, age, and/or household income, interactions terms between each biomarker index and each modifying variable were added to the logistic regression models. If the interaction term was significant (p < 0.10), the association between SRH and the biomarker index was deemed to be modified by the given variable. Stratified logistic regression models by gender, age (categorical), and household income (in tertiles) were then conducted to assess how the association between SRH and each biomarker index varied for women vs. men; working-age vs. retired-age adults; and high-income vs. middle-income vs. low-income groups.

## Results

Table 3 displays sample characteristics; the mean (SE) for each biomarker and biomarker index for the sample as a whole and by gender, age, and household income; and the associations between SRH and each biomarker and biomarker index. The sample was 56% female and over two-thirds were working-age (aged 25–60 years). For all biomarker indices, means varied by gender (women higher than men), age (retired-age higher than working-age), and household income (highest in the low-income, followed by the middle-income, and lowest in the high-income); however, these differences were small.

**Table 3:** Weighted characteristics of the sample; mean (SE) for each biomarker and biomarker index for the sample as a whole and by gender, age, and household income; and associations between self-rated health (SRH) and biomarkers based on logistic regression models.^a^

	N	Freq (%)	Total Mean (SE)	Gender		Age		Household income			Association with SRH OR (95%CI)
				Women Mean (SE)	Men Mean (SE)	25-60y Mean (SE)	≥60y Mean (SE)	Low Mean (SE)	Middle Mean (SE)	High Mean (SE)	
**Self-rated health**	15,687										
Good		78.84									
Bad		21.16									
Gender^b^	15,687										
Women		55.85									0.98 (0.90–1.07)
Men		44.15									1.00
**Age**^b^	15,687		51.11 (0.14)								
Working-age (25-60y)		68.06									1.00
Retirement-age (≥60y)		31.94									0.53 (0.49–0.58)
**Household income**^b^	15,687		1741.87 (19.31)								
Low		33.95									0.41 (0.37–0.46)
Middle		32.39									0.55 (0.41–0.63)
High		33.66									1.00
**VISIBLE WEIGHT-RELATED BIOMARKERS**	15,687										
Body Mass Index (kg/m^2^)			28.01 (0.05)	28.03 (0.07)	28.00 (0.06)	27.80 (0.06)	28.48 (0.07)	28.27 (0.09)	28.19 (0.08)	27.58 (0.07)	0.93 (0.93–0.94)
Weight status		0.77									
Underweight		29.91									0.49 (0.30–0.82)
Normal weight		38.85									1.00
Overweight		30.47									0.86 (0.77–0.97)
Obesity											0.44 (0.39–0.49)
Waist circumference (cm)			94.07 (0.12)	89.88 (0.16)	99.37 (0.17)	92.51 (0.16)	97.38 (0.18)	94.74 (0.22)	94.47 (0.21)	93.01 (0.20)	0.97 (0.96–0.97)
Abdominal obesity (AO)^c^		30.42									
No AO		24.40									1.00
At risk of AO		45.18									1.00 (0.87–1.14)
AO											0.47 (0.42–0.53)
%Body fat (%BF)		37.66	30.97 (0.10)	36.66 (0.10)	23.77 (0.12)	30.31 (0.12)	32.38 (0.15)	31.75 (0.18)	31.18 (0.16)	29.98 (0.16)	0.97 (0.96–0.97)
Obesity based on %BF^d^											0.71 (0.64–0.78)
Visible index^e^			5.92 (0.02)	5.95 (0.03)	5.88 (0.03)	5.70 (0.02)	6.38 (0.03)	6.06 (0.03)	5.99 (0.03)	5.71 (0.03)	0.84 (0.82–0.86)
**FITNESS BIOMARKERS**	10,636										
Heart rate (bpm)			68.80 (0.11)	69.91 (0.14)	67.45 (0.17)	69.22 (0.14)	67.91 (0.18)	69.63 (0.20)	68.76 (0.19)	68.06 (0.18)	0.98 (0.98–0.99)
Grip strength (kg)			34.28 (0.12)	26.97 (0.10)	43.15 (0.16)	36.43 (0.16)	29.81 (0.18)	32.57 (0.22)	34.33 (0.22)	35.84 (0.21)	1.05 (1.04–1.06)
FEV1%			92.40 (0.17)	93.39 (0.23)	91.21 (0.26)	93.49 (0.19)	90.12 (0.35)	90.03 (0.33)	92.34 (0.29)	94.68 (0.26)	1.02 (1.02–1.03)
FVC%			97.37 (0.16)	98.13 (0.22)	96.43 (0.23)	97.62 (0.19)	69.84 (0.31)	95.76 (0.31)	97.18 (0.27)	99.05 (0.25)	1.02 (1.02–1.03)
Fitness index^e^			7.93 (0.02)	7.92 (0.03)	7.94 (0.03)	7.70 (0.03)	8.41 (0.03)	8.29 (0.04)	7.93 (0.04)	7.60 (0.04)	0.80 (0.73–0.82)
**FATIGUE BIOMARKERS**	9,321										
C-reactive protein (mg/L)			2.09 (0.02)	2.23 (0.03)	1.93 (0.03)	1.94 (0.03)	2.37 (0.04)	2.35 (0.04)	2.13 (0.04)	1.81 (0.03)	0.86 (0.84–0.88)
Fibrinogen (g/L)			2.77 (0.01)	2.82 (0.01)	2.70 (0.01)	2.67 (0.01)	2.94 (0.01)	2.84 (0.01)	2.78 (0.01)	2.68 (0.01)	0.61 (0.55–0.69)
Hemoglobin (g/L)			137.32 (0.15)	130.48 (0.15)	145.49 (0.19)	138.01 (0.20)	136.03 (0.22)	137.75 (0.27)	137.24 (0.26)	137.94 (0.26)	1.01 (1.00–1.01)
Anemia^f^		10.30									0.75 (0.64–0.88)
Ferritin (μg/L)			133.80 (1.29)	92.54 (1.16)	183.04 (2.16)	126.08 (1.64)	148.42 (2.07)	127.68 (2.25)	131.54 (2.13)	141.79 (2.29)	1.00 (1.00–1.00)
CMV infection		51.7									0.84 (0.75–0.94)
Fatigue index^e^			8.04 (0.02)	8.10 (0.03)	7.97 (0.03)	7.86 (0.03)	8.39 (0.03)	8.28 (0.04)	8.10 (0.03)	7.76 (0.03)	0.84 (0.81–0.87)
**DISEASE RISK BIOMARKERS**	6,847										
SBP (mmHg)			126.49 (0.20)	123.41 (0.29)	130.19 (0.29)	122.82 (0.25)	133.35 (0.34)	127.85 (0.41)	126.38 (0.35)	125.38 (0.34)	0.99 (0.98–0.99)
DBP (mmHg)			73.64 (0.13)	72.74 (0.18)	74.71 (0.21)	74.31 (0.18)	72.39 (0.21)	73.16 (0.25)	73.72 (0.23)	74.00 (0.22)	0.99 (0.98–0.99)
FEV1%			92.76 (0.21)	93.28 (0.29)	92.13 (0.31)	93.91 (0.24)	90.60 (0.42)	90.25 (0.41)	92.69 (0.36)	95.08 (0.33)	1.02 (1.02–1.02)
FVC%			97.92 (0.19)	98.21 (0.27)	97.56 (0.28)	98.17 (0.23)	97.44 (0.36)	96.24 (0.38)	97.67 (0.33)	99.66 (0.30)	1.02 (1.02–1.03)
Total cholesterol (mmol/L)			5.43 (0.01)	5.46 (0.02)	5.39 (0.02)	5.42 (0.02)	5.44 (0.02)	5.38 (0.03)	5.39 (0.02)	5.50 (0.02)	1.16 (1.09–1.23)
HDL cholesterol (mmol/L)			1.56 (0.01)	1.68 (0.01)	1.41 (0.01)	1.55 (0.01)	1.59 (0.01)	1.54 (0.01)	1.53 (0.01)	1.60 (0.01)	2.01 (1.67–2.42)
Triglycerides (mmol/L)			1.74 (0.01)	1.55 (0.01)	1.96 (0.02)	1.71 (0.02)	1.78 (0.02)	1.76 (0.02)	1.75 (0.02)	1.71 (0.02)	0.82 (0.77–0.88)
HbA1c (mmol/mol)			36.84 (0.08)	36.66 (0.11)	37.05 (0.13)	35.40 (0.09)	39.52 (0.13)	37.86 (0.16)	36.79 (0.14)	35.97 (0.11)	0.95 (0.94–0.96)
Disease index^e^			16.03 (0.04)	16.11 (0.06)	15.93 (0.06)	15.62 (0.06)	16.80 (0.06)	16.44 (0.07)	16.09 (0.07)	15.60 (0.07)	0.89 (0.87–0.91)

a Logistic regression models predicting reporting a “good” health and adjusted for age, gender, and household income (in tertiles).

b Associations between SRH and gender, age and household income were examined with a logistic regression model with SRH as the dependent variable and gender, age, and income as independent variables (all in one model).

c WC > 102 cm for men and >88 cm for women = abdominal obesity; WC between 94 and 102 cm for men and between 80 and 88 cm for women = at risk of abdominal obesity; WC < 94 for men and WC < 80 for women = normal.

d %BF>30% for women and >25% for men = obesity; %BF ≤ 30% for women and %BF ≤ 25% for men = no obesity.

e Biomarker indices are operationalized as continuous variables with higher values reflecting the worse outcomes in each particular category. The visible index ranges from 3 to 9; the fitness and fatigue indices from 4 to 12; and the disease index from 8 to 24.

f Hemoglobin (Hb) < 130 g/L for men and Hb < 120 g/L for women = anemia; Hb ≥ 130 g/L for men and Hb ≥ 120 g/L for women = no anemia.

Overall, we found that all the biomarker group indices were negatively associated with SRH, with higher indices (reflecting worse health outcomes) associated with lower odds of reporting good health. The *fitness* index was more strongly associated with SRH than the other indices: a one-unit increase in the *fitness* index (i.e. worse fitness outcomes) was associated with 20% lower odds of reporting good health (Table 3). Following were the *visible* and the *fatigue* indices: a one-unit increase in either of the two indices was associated with 16% lower odds of reporting good health. On the other hand, a one-unit increase in the *disease risk* index was associated with only 11% lower odds of reporting good health.

Almost all the individual biomarker-SRH associations were significant and in the expected direction (e.g. higher BMI, waist circumference, and %body fat associated with lower odds of reporting good health). Only ferritin was not significantly associated with SRH, whereas total cholesterol was associated with SRH in the unexpected direction (higher total cholesterol, higher odds of reporting good health). Total cholesterol includes both LDL and HDL cholesterol, though, and HDL is positively associated with SRH, driving the relationship of total cholesterol and SRH in the positive direction as well. The *visible* individual biomarkers were more strongly associated with SRH compared to the other biomarkers. For example, the odds of reporting good health among the obese (defined based on BMI, Table 1) were 56% lower compared to those with a normal weight (Table 3).

**Fig. 1:**
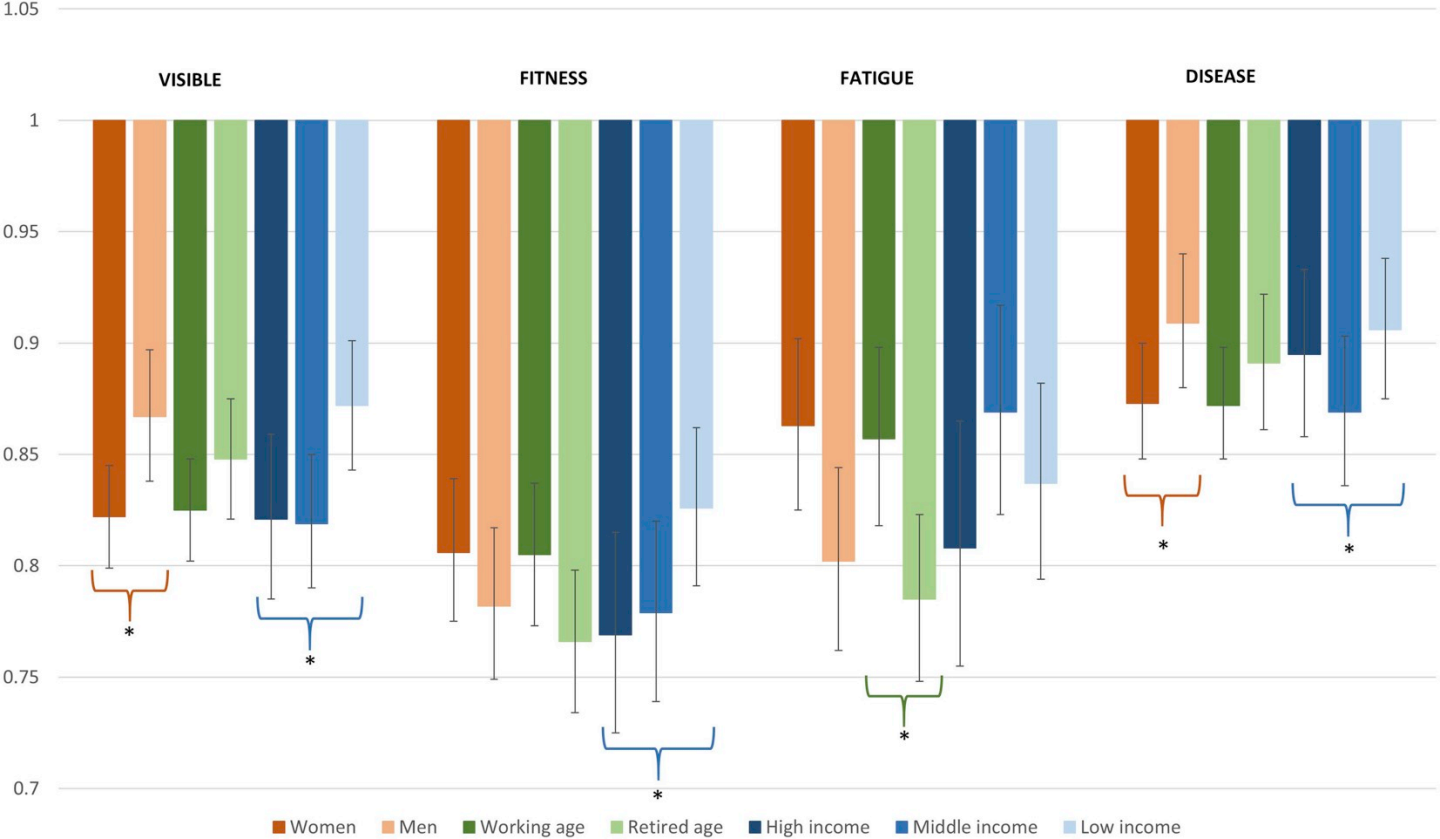
**Effect modification by gender, age, and household income of the association between self-rated health and biomarker groups indices.** Note: The asterisk (*) denotes a significant modifying effect, based on interaction terms added between the index and the modifying variables under study. Displayed odds ratios and 95% confidence intervals are based on stratified logistic regression analyses by gender, age, and household income groups.

Fig. 1 shows the results from the stratified analysis, displaying moderation effects by gender, age, and household income in the association between SRH and each biomarker group index. In terms of moderation by gender, we found that the association between SRH and the *visible* index was significantly stronger for women than men (interaction for *visible* index*gender p-value = 0.0088). Upon gender stratification, we found that a one-unit increase in the *visible* index was associated with 18% lower odds of reporting good health for women vs 13% lower odds for men. We found a similar gender modification for the *disease risk* index (interaction p-value = 0.0799): a one-unit increase in the *disease risk* index was associated with 13% lower odds of reporting good health for women vs. 9% lower odds for men (Fig. 1). For the *fitness* and *fatigue* indices, the association between SRH and these indices seemed stronger for men than women in magnitude, but the gender interactions were not significant (p-value = 0.3548 and p-value = 0.1203, respectively).

In terms of age moderation, we found that the association between SRH and the *fatigue* index was significantly stronger for the retired-age group, compared to those in the working-age group (interaction p-value for *fatigue* index*age = 0.0176). A one-unit increase in the *fatigue* index was associated with 22% lower odds of reporting good health for those in the retired-age group compared to 14% lower odds in the working-age group (Fig. 1). No significant age modification was found for the other biomarker indices.

We observed income moderation for the *visible, fitness* and *disease risk* indices, with stronger associations with SRH in the high-income vs. the low-income groups (interaction p-value = 0.0013, p-value = 0.0102, and p-value = 0.0854, respectively). A one unit increase in the *visible, fitness* and *disease risk* indices was associated with 18%, 23%, and 11% lower odds of reporting good health for those in the high-income group, compared to 13%, 17%, and 9% lower odds for those in the low-income group, respectively. In addition, for the *disease risk* index, those in the middle-income group had a significantly stronger association between *disease risk* index and SRH (13% lower odds of reporting good health) vs. those in the high-income group (11% lower odds; interaction p-value = 0.0811).

## Discussion

The aim of this study was to investigate the association between SRH and a range of biomarkers in order to understand differential reporting of SRH in social surveys. We grouped biomarkers into four categories (*visible, fitness, fatigue,* and *disease risk*) and examined whether their association with SRH was modified by gender, age, and income among a sample of adults in Britain. We found that SRH was associated with the majority of the biomarkers under study in the expected direction, with the *fitness* index having the largest impact on the way people rate their health. The *visible* biomarkers, individually, also had strong associations with SRH. Moreover, we found some modification effects by gender, age, and income, with mixed results regarding support for our hypotheses.

Our finding that SRH was significantly associated with objectively measured biomarkers has support in the literature (Christian et al., 2011; Jarczok et al., 2015; Jylhä et al., 2006; Saudny, Cao, & Egeland, 2012; Undén et al., 2007). Jylhä et al. (2006) found that SRH was significantly associated with albumin, hemoglobin, white cell count, HDL-cholesterol, and creatinine among a sample of older adults in three U.S. states. Similarly, Saudny et al. (Saudny et al., 2012) found that waist circumference, triglyceride levels, and CRP were associated with SRH among a sample of Canadian Inuit. Jarczok et al. (Jarczok et al., 2015), on the other hand, found that heart rate variability was the only biomarker related to SRH among a convenience sample in Southern Germany; they found no association between SRH and inflammatory markers, blood pressure, and blood lipids. However, our paper goes beyond these previous analyses by trying to conceptualize biomarker groups as reflecting different dimensions of health and evaluating how these different health dimensions might be associated with self-ratings of health.

Our finding that the *visible* biomarkers were strongly associated with SRH compared to the other biomarker types could be explained by the current abundance of health messages related to obesity and its negative consequences (Altman et al., 2016). People's exposure to obesity-related health messages may not have an effect on their actual body weight, but it may influence their knowledge and social norms regarding body weight and thus influence the way they rank their health (Medic et al., 2016). Similar conclusions could be reached regarding the *fitness* biomarkers, with strong media emphasis on fitness for health.

Alternatively, individuals with a high value for the *visible* index would have either a high BMI, a high waist circumference, a high % body fat, or all the above. These high values in body mass or body fat indicators could also translate into worse objective health outcomes, as obesity is strongly associated with numerous chronic conditions including heart disease and diabetes. If this was the case, the presence of diseases associated with obesity among these individuals could be influencing the way they rate their health and driving the observed association between the *visible* index and SRH. Still, we believe that the social component of the *visible* index would be the most likely explanation here since the association between the *visible* index and SRH is stronger in magnitude than the association between the *disease risk* index and SRH, and all of the indicators included in the *disease risk* index would be those affected by obesity.

As for our findings regarding moderation, we tested 12 different moderation effects and found that only six of these were significant. We proposed 10 specific hypotheses in reference to these 12 moderation effects and found support for only four of them. This may imply that the association between SRH and biomarker groups do not vary by gender, age, and income to the extent hypothesized. In terms of gender, we hypothesized that the association between SRH and biomarkers for women vs. men would be stronger for the *visible* biomarkers and weaker for the *fitness* biomarkers, only the first of which we found to be true. Blaxter (1990) reported that women often describe someone healthy in terms of *being or feeling slim,* whereas men are more likely to define health in terms of *physical strength*. There are reports that women are more often discriminated against because of their weight than men (Puhl, Andreyeva, & Brownell, 2008), and are more aware of the negative health consequences of obesity (Gregory, Blanck, Gillespie, Maynard, & Serdula, 2008). Therefore, it is reasonable to assume that women would be more conscious about *visible* biomarkers when rating their health than men. On the other hand, Okosun et al. (Okosun, Choi, Matamoros, & Dever, 2001) found that, among a representative sample of U.S. adults, the association between obesity and SRH was lower among women than men, with the exception of those who identified themselves as Hispanic. It is important to note that even though we did not find significant differences for men vs. women in the *fitness* index (interaction p-value = 0.3548), the relationship between the *fitness* index and SRH was stronger for men (OR = 0.782, 95%CI = 0.749–0.817) than women (OR = 0.806, 95%CI = 0.775–0.839).

We also hypothesized that the association between SRH and *fatigue* biomarkers would be stronger among women than men, but we found no significant gender moderation for the *fatigue* index and a trend towards the opposite direction. Blaxter (1990) reported that *energy* was the word most frequently used by all women when describing health, and a population study in western Sweden found that tiredness/weakness was the main symptom associated with prevalence of poor SRH, with the association being more pronounced among women than men, particularly among working-age adults (compared to those >65 years) (Molarius & Janson, 2002). It is possible that the biomarkers we chose to represent *fatigue* were not adequate enough to capture this concept of “energy” or that the biomarker values in our sample were not extreme enough to cause any fatigue-related symptoms. However, a recent publication also based on the UKHLS sample confirmed an association between CRP (one of the components of the *fatigue* index) and self-reported fatigue, based on the question *“How much of the time during the past 4 weeks did you have a lot of energy?*” contained in the Short Form Health Survey (SF-12) (Hughes & Kumari, 2017). They also found no gender interaction in this association, but a strong interaction for age, as discussed below.

In terms of age, we hypothesized that the association between SRH and *fitness* biomarkers would be weaker for the retired-age group compared to working-age adults. We found a trend in the opposite direction (Fig. 1), but it was not statistically significant (interaction p-value = 0.1315). Our assumption was that older respondents may lower their expectations about their fitness levels compared to their younger counterparts. However, it may be that we did not choose a high enough age cut-off (60 years) for such expectations, that more current cohorts (i.e. those born in the 1950s rather than 1920s) than those studied by Blaxter in the 1980s do not share the same reduced expectations, or that the biomarkers included in the *fitness* index were not sufficiently different between the working-age and the retire-age groups to influence the way they make people feel and, hence, affect the way they rate their health. Blaxter (1990) reported that *physical fitness* was the least favored concept to define health for oneself or others for participants over 60 years, while stating that *energy* was an important consideration for older men when rating their health, a report that was supported by our finding that the association between SRH and the *fatigue* index was stronger for the retired-age group compared to working-age adults. This finding also has support in the abovementioned study comparing CRP and self-rated fatigue among UKHLS participants (Hughes & Kumari, 2017); in that study, CRP was predictive of future self-reported fatigue among older (>60 years) participants but not among those in the younger groups.

We also hypothesized that the association between the *disease risk* biomarkers and SRH would be weaker for the retired-age group compared to those in the working-age group, for which we did not find support. This result contradicts our hypothesis that older people are less likely to consider chronic conditions when rating their own health since the presence of disease may be more common in this group, particularly when they compare themselves against age peers (Blaxter, 1990). The lack of such an association may, as above, reflect different age cut-offs or changing expectations with newer cohorts or the fact that our *disease risk* index was not sensitive enough to capture those with disease conditions and/or symptoms, which would influence health ratings. Jylhä, Leskinen, Alanen, Leskinen, and Heikkinen (1986) found that one of the best predictors of SRH among 71-75-year-old Finnish men were the number of chronic diseases they suffered. Jylhä also noted in a later publication that being free of serious disease is the baseline for health assessment of younger people, whereas the evaluation context is more complex among the elderly, with most old people negotiating “between the normative non-problematic category of ‘good’ health and experienced problems in health and functioning” (Jylhä, 2009). Along these lines, Heller, Ahern, Pringle, and Brown (2009) found that an increase in comorbidity over time worsened SRH, but the impact of comorbidity change was less pronounced among older individuals.

We found mixed support for our hypotheses regarding income moderation. For the *visible* index, we hypothesized that the association with SRH would be stronger among those in the high-income group compared to less affluent participants, which was the case (Fig. 1). There are documented income inequalities in obesity prevalence, with those living in the lowest income brackets having higher rates of obesity (Booth, Charlton, & Gulliford, 2017). Therefore, our hypothesis that the association between SRH and *visible* biomarkers will be stronger among those in the highest income groups seemed appropriate given the lower obesity prevalence and the heightened weight-related social norms in this group (Wardle & Griffith, 2001).

We did not find support for our hypotheses of stronger SRH-*fitness* and *fatigue* indices associations for lower income groups; on the contrary, the association between the *fitness* index and SRH was stronger among those in the highest income tertile compared to those in the lowest. It is possible that income is not the most appropriate measure of SEP to investigate the hypothesized importance of *fitness* in low socioeconomic groups. However, when using education instead of income in our sample, we found no significant moderation between SRH and the *fitness* index (data not shown). Blaxter (1990) found that among working-age men, health was often defined as “being able to do hard work,” particularly among those who did manual work (Blaxter, 1990). However, the importance of manual labor in the UK has been decreasing since the 1990s following deindustrialization, so Blaxter's results may not be as relevant today. In fact, being physically fit in terms of body size and composition (i.e. being slim or having low body fat), as opposed to “being able to do hard work” may be more relevant in current times, in particular among high SEP groups (Wardle & Griffith, 2001).

As we hypothesized, we observed that income moderated the association between SRH and the *disease risk* index, with stronger associations among those in the high-income group. Blaxter (1990) reported that health as *not being ill* was more commonly mentioned for those with higher education and higher incomes. In addition, other research suggests that less educated groups may have lower expectations, as the peers they compared themselves against when ranking their own health have a higher-than-average prevalence of disease, hence this may impact less on their self-assessment (Bago d’Uva, O'Donnell, & van Doorslaer, 2008). Though it is important to note the *disease risk* index is based on underlying blood analytes, and not on whether someone has been diagnosed with a health condition.

This study has several strengths and limitations. Our sample is based on a large representative study of residents of Great Britain, with a diverse sample in terms of age and income, allowing us to explore associations between SRH and biomarkers across life stages and socioeconomic groups. Data on a variety of biomarkers allowed us to categorize these biomarkers into groups based on their function or likely symptoms for a more in-depth analysis of the association between SRH and objective health outcomes than has been explored in the literature to date. However, the groupings were decided upon by the authors for the purposes of this analysis, and therefore contain a subjective element. It is possible that different groupings would have led to different results. In addition, our focus on gender, age, and income moderation of the association between SRH and objective health is novel. However, we investigated effect modification by gender, age, and income *individually*, without accounting for possible interactions between them. It is likely that none of the demographic factors of importance (age, gender, income, etc.) operate in isolation to affect the relationship between objective health and SRH; therefore, future studies should incorporate an intersectionality perspective to study the nuances associated with SRH responses (Brown, Richardson, Hargrove, & Thomas, 2016; Reczek, Liu, & Spiker, 2017; Veenstra, 2011). Our study did not incorporate other possible influential factors such as race/ethnicity (e.g. Allen, McNeely, & Orme, 2016) or sexual orientation (e.g. Veenstra, 2011). Even though UKHLS includes a minority boost sample, this sample was not included in the nurse visit and, therefore, our analysis was primarily based on White/European (97%), impeding us from carrying out analysis for racial/ethnic minorities. Finally, there are no biomarkers reflecting psychological health, but previous research highlights the frequency with which non-physical factors (i.e. attitudes, emotions) are used to describe health (Benyamini et al., 2000; Blaxter, 1990; Borawski, Kinney, & Kahana, 1996; Jylhä, 2009). Future studies should examine objective measures of both physical and psychological health in relation to SRH, and investigate if the importance of each varies by age, gender, SEP, and/or race/ethnicity.

In conclusion, while SRH is overall strongly associated with objective measures of health, we found that the strength of this association varies by the type of biomarker used as well as by gender, age, and income, though the latter to a lower extent than we hypothesized. While SRH is a valuable health indicator, caution should be taken when using SRH as the sole health measure when studying gender, age, and income health inequalities.

## Ethical statement

Approval from the National Research Ethics Service was obtained for data collection (Oxfordshire A REC, Reference: 10/H0604/2).

## Declaration of interest

None.
